# Widespread Disulfide Bonding in Proteins from Thermophilic Archaea

**DOI:** 10.1155/2011/409156

**Published:** 2011-09-20

**Authors:** Julien Jorda, Todd O. Yeates

**Affiliations:** ^1^UCLA-DOE Institute for Genomics and Proteomics, 611 Charles Young Drive East, Los Angeles, CA 90095, USA; ^2^Department of Chemistry and Biochemistry, University of California, Los Angeles, 611 Charles Young Drive East, Los Angeles, CA 90095, USA

## Abstract

Disulfide bonds are generally not used to stabilize proteins in the cytosolic compartments of bacteria or eukaryotic cells, owing to the chemically reducing nature of those environments. In contrast, certain thermophilic archaea use disulfide bonding as a major mechanism for protein stabilization. Here, we provide a current survey of completely sequenced genomes, applying computational methods to estimate the use of disulfide bonding across the Archaea. Microbes belonging to the Crenarchaeal branch, which are essentially all hyperthermophilic, are universally rich in disulfide bonding while lesser degrees of disulfide bonding are found among the thermophilic Euryarchaea, excluding those that are methanogenic. The results help clarify which parts of the archaeal lineage are likely to yield more examples and additional specific data on protein disulfide bonding, as increasing genomic sequencing efforts are brought to bear.

## 1. Introduction

The archaea inhabit incredibly diverse environments [1]. Many species thrive at temperatures exceeding 100°C. Growth at such high temperatures presents special challenges, among the most serious being the problem of stabilizing cellular proteins in their natively folded configurations. For many proteins, the folded configuration is only modestly favored energetically compared to the unfolded state [2], and high temperatures irreversibly unfold the vast majority of proteins derived from organisms that live at moderate temperatures. The question of how thermophilic proteins are stabilized has therefore attracted considerable attention over the years [3, 4].

Numerous studies have concluded that thermophilic proteins are stabilized by a wide array of forces and effects, which appear to present themselves to different degrees in different proteins and organisms [3, 5–8]. Increased atomic packing [9, 10], hydrophobic interactions [11], ionic interactions [9, 12–14], and shorter loops [15] have all been noted as providing additional noncovalent stabilization in thermophilic proteins. More unexpected was the realization that disulfide bonding—a much stronger, covalent force—might play an important role in some organisms [16, 17]. A striking clue came when the structure of the enzyme adenylosuccinate lyase from the hyperthermophilic *Pyrobaculum aerophilum* revealed that the six cysteines in the protein chain pair up to form three disulfide bonds [17]. This prompted the development by Mallick et al. [16] of genomic calculations, which supported the idea that some thermophiles use disulfide bonding as a major mechanism for protein stabilization [16, 18, 19]. Subsequent proteomic experiments on *P. aerophilum* validated that claim [20], as have recently published structures [21–23] and biochemical studies [24–26] of proteins from various hyperthermophilic archaea.

The use of disulfide bonding came as a surprise, because in well-studied organisms the intracellular environment is chemically reducing, and therefore favors the thiol form of cysteines over the disulfide form (reviewed in [27]). Though disulfide bonds are a common mechanism for stabilizing proteins that are either secreted or reside in oxidizing extracytosolic compartments, thermodynamic considerations prevent disulfide bonds from conferring protein stability under reducing conditions. This is a general rule, notwithstanding the existence of varied cytosolic proteins that form disulfide bonds transiently or reversibly, as part of cellular redox signaling mechanisms, for example [28–30]. The prevalent use of disulfides therefore brought up new questions about the intracellular environments of archaea, and the molecular mechanisms for forming protein disulfide bonds within the cytosol. Comparative genomics studies showed that a protein known as protein disulfide oxidoreductase (PDO) was present in thermophiles, and selectively in organisms predicted to be rich in intracellular disulfide bonding [18, 31]. This helped focus attention on PDO as the presumptive key player in intracellular protein disulfide bonding (reviewed in [32–34]), a role that is consistent with in vitro studies on PDO from multiple thermophiles [35–37].

The importance of disulfide bonding in thermophiles emerged when complete genomes were known for only about 25 unique prokaryotes, of which seven were archaea [16]. There are presently 1031 completely sequenced prokaryotic genomes with accompanying proteomes available at the National Center for Biotechnology Information web server. Though archaeal species constitute an unfortunately small fraction of this set—90 out of 1031—their growing number provides an opportunity for an updated assessment of thermophilic protein disulfide bonding in this important and diverse branch of the tree of life.

## 2. Results and Discussion

Protein sequences from 90 complete archaeal proteome sets were obtained from the UniProt web server, release 2011-4. Sequences were also retrieved for several viruses infecting *Sulfolobus* species, and viruses infecting *Pyrobaculum aerophilum*, *Pyrococcus abyssi*, and *Thermoproteus tenax*, along with data for five moderately thermophilic eubacteria: *Thermotoga maritima*, *Aquifex aeolicus*, *Streptococcus thermophilus, Thermosipho melanesiensis, *and* Thermobaculum terrenum*. In addition, metagenomic sequence data from archaeal-rich microbial communities sampled at geothermal springs in Yellowstone National Park were obtained from Inskeep et al. [38].

**Figure 1:**
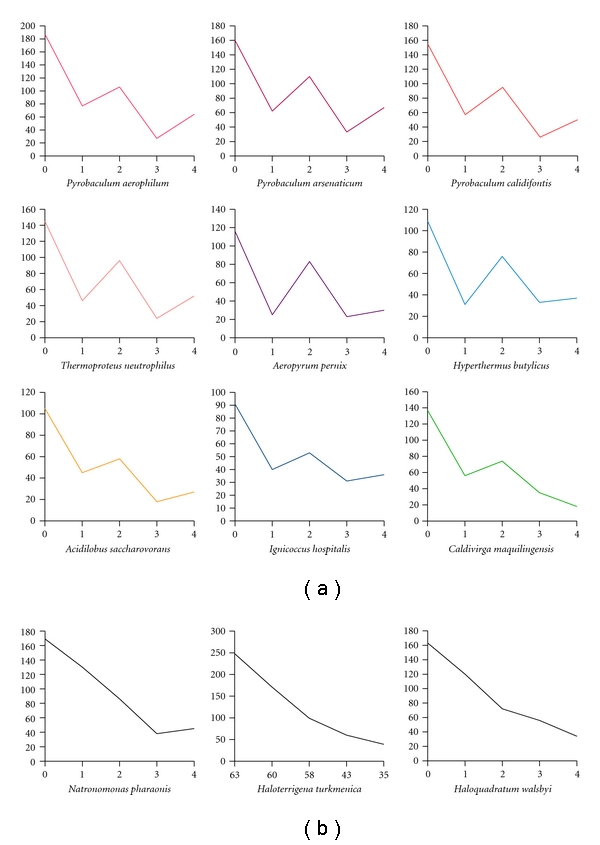
A preference for an even versus odd number of cysteines in proteins from thermophilic archaea. The dataset used for these plots consists of proteins with sizes ranging from 150 to 200 amino acids, the expected trend being more apparent for this class of proteins. (a) Numerous hyperthermophilic and thermophilic archaea show a clear propensity for even numbers of cysteine residues. This trend suggests an abundance of disulfide bonds. Nine examples are shown. (b) Selected nonthermophilic species (all halophiles) are shown as controls. In these cases, the plotted lines are nearly monotonic, indicating an absence of significant disulfide bonding in the halophiles.

We applied both of the methods introduced by Mallick et al. [16] for analyzing disulfide bonding in the present study. The first is based on an enumeration of proteins having an even versus an odd number of cysteine residues. If an organism has a strong tendency for all or most of its cysteines to be paired into disulfide bonds, then one can expect to see an overrepresentation of proteins with an even number of cysteines. Indeed, such a trend is clear for several of the hyperthermophilic archaea examined (Figure 1). No such preference is seen in control calculations involving nonthermophilic bacterial genomes, as long as care is taken to filter out proteins destined for export from the cytosol; the formation of disulfide bonds in the oxidizing environment of the bacterial periplasm has been extensively studied, and in fact an analysis of even versus odd cysteines has been used to illustrate the abundance of disulfide bonds in secreted proteins or those destined for the bacterial periplasm [39, 40]. Although the simple cysteine counting approach gave clear results in the present study for a number of hyperthermophiles, the pattern was less clear for some organisms, including some of the *Sulfolobus* species, despite earlier data indicating that disulfides should be abundant [18]. It is notable that some archaea, including *Sulfolobus*, are relatively rich in metalloproteins [41], whose metal sites are often coordinated by cysteines, potentially hampering an accurate prediction of disulfide abundance based on a simple counting of cysteine residues. This prompted an alternate analysis based on sequence-structure mapping.

**Figure 2:**
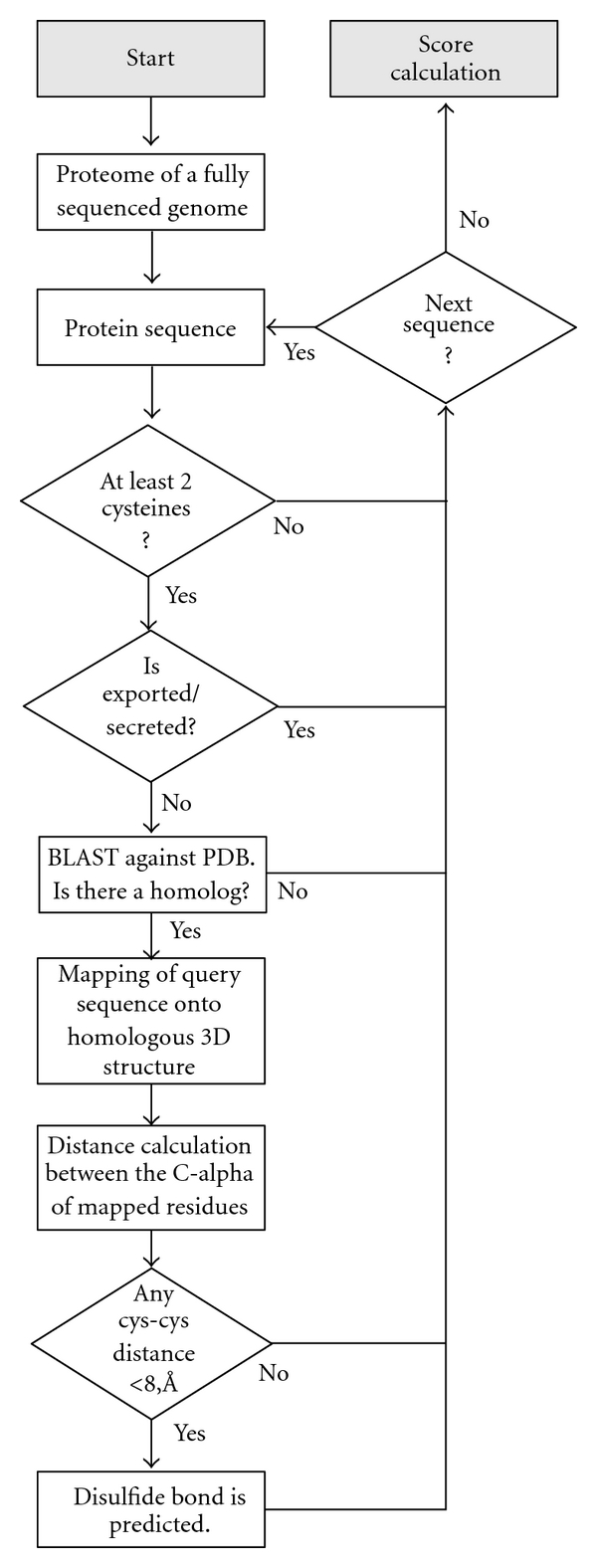
Flowchart illustrating the procedure for mapping genomic sequence data onto known three-dimensional protein structures in order to estimate disulfide abundance. Grey boxes are the starting and ending points of the pipeline, white boxes are processing steps, and diamonds are decisions made subsequent to filtering steps. The procedure loops until all the proteins from a given proteome are processed.

A second method for disulfide bond analysis relies on the availability of known three-dimensional protein structures, not of the specific genomic proteins in question, but of homologous proteins from other organisms (Figure 2). The underlying principle is that if a given protein sequence (from a hyperthermophilic archaeon for example) has two cysteines that form a disulfide bond in the folded configuration, then when that query sequence is mapped or overlaid onto the structure of a homologous protein, the two cysteines in the query sequence should be nearby in space, as would be required if a bond between them was present [16]. The specific value, *f*, that we report in the present study is the fraction of all cysteine residues (among those (*m*) that could be mapped onto structures) that fall within 8 Å (C-alpha to C-alpha) of some other cysteine residue in the modeled structure. We refer to this fraction, *f*, as the predicted disulfide abundance parameter. Whereas the cysteine counting method suffers from the oversimplified notion that all the cysteines in a protein must be disulfide bonded, the sequence-structure mapping method presents other difficulties. Only partial coverage can be expected, since many query protein sequences will not be represented by homologous structures in the PDB. In addition, in cases where a homologous structure is available, substantial evolutionary divergence between the query and the target protein can lead to unreliable alignments as well as to bona fide structural differences, both of which tend to reduce the likelihood that the cysteines will appear in close proximity as required. Nonetheless, the method provides the advantage of specificity in three dimensions.

**Figure 3:**
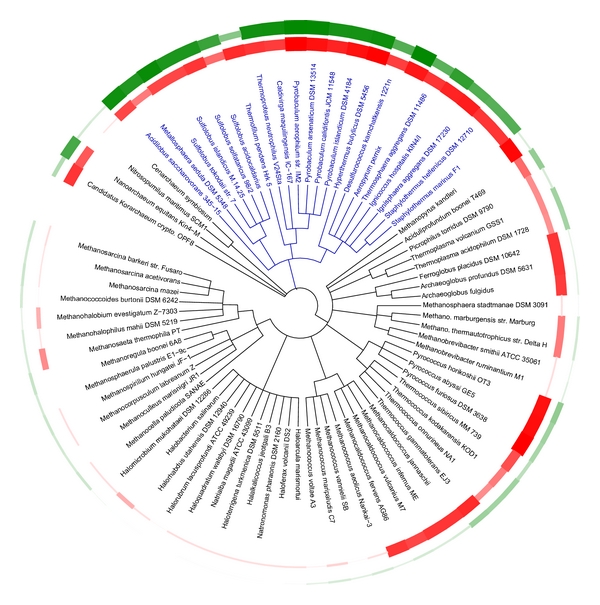
A phylogenetic tree showing the predicted abundance of disulfide bonding across the archaea. Each leaf of the tree is associated to specific values of the calculated disulfide abundance parameter (*f*) and optimal growth temperature, which are depicted in respective circular plots, green (outer ring) and red (inner ring). These values are represented by the properties of the curves, which vary in thickness (and darkness) in proportion to the corresponding quantity. A general correlation is seen between disulfide richness and growth temperature; specifically, disulfide bonding is dominant in the Crenarchaea (blue) and also notable in the subset of thermophilic Euryarchaea that are nonmethanogenic.

When applied to the collection of genomic data, the sequence-structure mapping method provides a clear indication of disulfide richness for many of the thermophilic archaea (Figure 3; see Table 1-Supplementary Material available online at doi:10.1155/2011/409156). This is also the case for the *Sulfolobus* species noted above, whose analysis had been unclear by the simple cysteine counting method. In all, roughly 33 archaeal genomes (not counting closely related strains of the same genus) are judged to have significant amounts of disulfide bonding (*f* > 0.15) while smaller subsets show even higher values (21 genomes with *f* > 0.25, and 8 with *f* > 0.35) (See Supplementary Table S1). In addition, the unusual, moderately thermophilic eubacteria have a detectable but lower fraction of their cysteines in disulfide bonds than archaeal thermophiles, with the exception of *Aquifex aeolicus,* which stands out among this group. This result is in accordance with previous studies highlighting that *Aquifex *has a higher fraction of proteins in common with archaeal thermophiles than any other nonthermophilic eubacteria [42]. As a final source of thermophilic sequences, we performed an initial analysis, which should be considered preliminary (see Section 4), of metagenomic sequence data derived from archaeal-rich hot springs in Yellowstone National Park [38]. Overall, these data showed strong evidence for disulfide bonding. For the cellular DNA sample from Nymph Lake (site 10, August 2009), *f* was 0.29 while for the sample from Crater Hills (September 2009), the value was 0.35, which is comparable to the highest values obtained for individually sequenced genomes so far.

**Figure 4:**
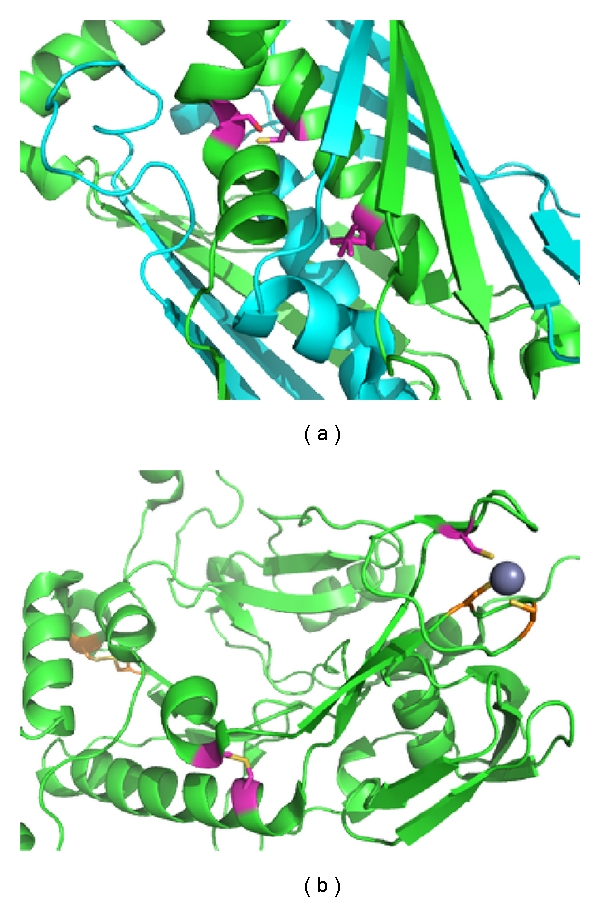
Examples of known thermophilic archaeal protein structures containing disulfide bonds, but having an odd number of total cysteines. Two proteins from *Sulfolobus islandicus *M.14.25 are shown. Mapped cysteines positions are represented in pink while cysteine residues involved in a putative metal-binding site are in orange, Zn in grey. (a) Mapping of an OsmC family protein (UniProtKB : C3MY18) onto the PDB structure 2OPL. One disulfide bond is predicted; the third cysteine is located at the surface and could participate in an intermolecular disulfide bond. (b) Mapping of a probable DNA primase small subunit (UniProtKB : C3MYF5) onto the PDB structure 1ZT2. Two mapped cysteines are poised to form a disulfide bond while the third is close to a Zn-binding site, suggesting an interaction with the metal ion. Many cases of proteins with an odd number of cysteines probably reflect the presence of intermolecular disulfide bonds or participation in metal-binding sites.

Specific cases were investigated where disulfide bonding in a genome was evident by the sequence-structure mapping method, but was missed by the cysteine counting method. Several of the proteins from *Sulfolobus islandicus *strain M.14.25 that have exactly three cysteine residues were examined. Of the 771 sequences that could be mapped to a PDB homologue, 92 had three cysteines that could be mapped onto the structure. Among them, 53 were predicted to have a disulfide bond, and one cysteine thiol apart. Analysis of mapped structures showed that the free cysteine tended to be either involved in a putative metal-binding site or poised on the surface. The latter scenario is suggestive of potential intermolecular disulfide bonds. That hypothesis is strengthened by the observation that cases with a third, exposed cysteine occurred often in structures that were oligomeric. Two illustrative examples—one involving a likely intermolecular disulfide and one involving a likely metal binding site—are shown in Figure 4. These situations highlight a key difficulty with the cysteine counting method: proteins having an odd number of cysteines may actually represent positive cases of disulfide bonds. Furthermore, even in disulfide rich organisms where free (reduced) cysteine thiols are systematically diminished, odd numbers of cysteines may be present in proteins that form intermolecular disulfide bonds. Proteomic experiments on *P. aerophilum*, using oxidized versus reduced 2D SDS gel electrophoresis, have highlighted the abundance of intermolecular disulfide bonding in that organism [20]. Crystal structures of proteins from other thermophiles further support this point [23, 43, 44].

The predicted abundance of protein disulfides across the Archaea correlates strongly with phylogenetic divisions, but there are also notable trends within specific branches (Figure 3). Essentially all of the Crenarchaea show very high levels of protein disulfide bonding. In contrast, disulfide bonding occurs at lower levels, and more selectively, within the Euryarchaea. More specifically, the effect is practically absent from the halophiles, but present in some thermophilic Euryarchaea. The presence or absence of significant disulfide bonding within the thermophilic Euryarchaea appears to correlate most strongly with the absence of methanogenesis. That trend could reflect the incompatibility of disulfide bonding with either the redox potential required for reduction of oxidized carbon to methane, or with the oxidation-prone enzymes and cofactors that carry out methanogenesis [45]. Beyond the well-studied crenarchaeal and euryarchaea, genomes have been sequences for microbes representing three ancient or highly divergent archaeal branches (Figure 3). Two of these, *Nanoarchaeum equitans* and *Candidatus Korarchaeum cryptofilum*, are thermophilic; of the two, *N. equitans* shows the greater degree of disulfide bonding.

We also investigated several viruses that infect thermophilic archaea. A nonexhaustive list of analyzed viruses is reported. The total number of cysteines that could be mapped onto structures (*m*) is provided along with the estimated disulfide abundance parameter (*f*): *Sulfolobus islandicus* rod-shaped virus 1 (*m* = 16, *f* = 1), *Sulfolobus islandicus* filamentous virus (*m* = 12, *f* = 0.75), *Sulfolobus* virus 1 (*m* = 18, *f* = 1), *Sulfolobus* virus Kamchatka (*m* = 14, *f* = 0.71), *Sulfolobus* turreted icosahedral virus (*m* = 14, *f* = 1), *Sulfolobus* virus Ragged Hills (*m* = 4, *f* = 1), *Pyrobaculum* spherical virus (*m* = 35, *f* = 0.57), *Sulfolobus* virus STSV1 (*m* = 12, *f* = 0.33), and *Sulfolobus* spindle-shaped virus 4 (*m* = 4, *f* = 1). The presence of disulfides in the *Sulfolobus* viruses has already been emphasized in reported crystal structures and by way of cysteine counting [46–48]. It is interesting that of the viruses that infect disulfide-rich thermophilic archaea, most appear to encode proteins that contain disulfide bonds. The results on individual viral genomes should be interpreted with caution, however, as most of the reported viruses have been characterized from the *Sulfolobus* species, and the total number of proteins encoded by a single virus is small, making statistically significant conclusions difficult.

**Figure 5:**
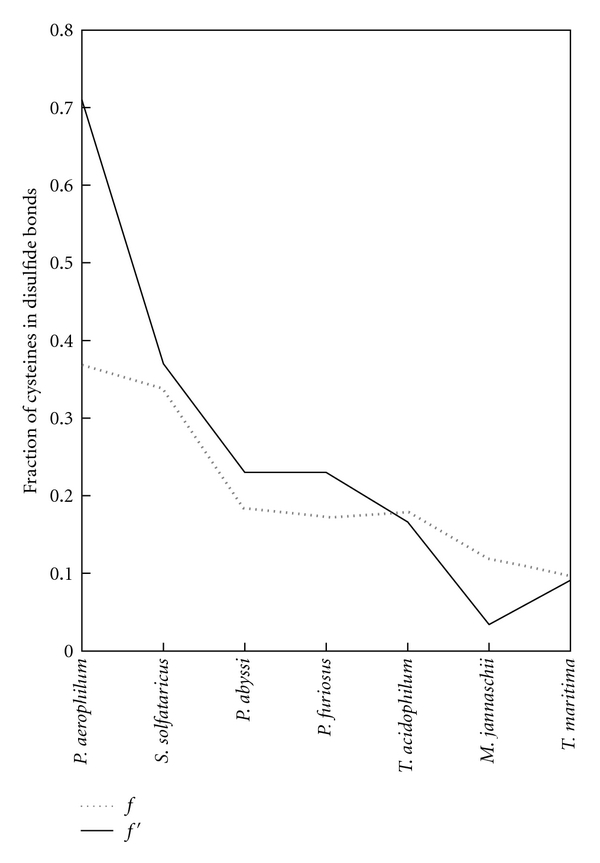
Correlation of the predicted disulfide abundance parameter, *f*, with the corresponding value *f*′ determined from the protein structure databank for thermophiles with significant coverage. Selected thermophiles were analyzed if they had sufficient representation in the PDB to obtain a reasonable estimate of the disulfide abundance. Metalloproteins were excluded. See Section 4.

For reasons already noted, the computational methods discussed give only rough measures of the true disulfide abundance in the proteins from a given genome. The errors and deviations arising during sequence-structure comparison and the challenge in interpreting intermolecular disulfide bonds both tend to cause an underestimation of the actual disulfide abundance; even if all the cysteines in the proteins from some genome were disulfide bonded, only a fraction would be successfully identified by the sequence-structure mapping approach. Fortunately, a few thermophilic and hyperthermophilic microbes have been popular targets for structural studies. Cases where many protein structures have been reported from a single organism present an opportunity to evaluate the disulfide abundance directly, assuming that the structures reported are reasonably representative of the genome as a whole. Examining the deposited protein structures from several selected organisms, we evaluated directly what fraction of the cysteines were involved in disulfide bonds. We denote this fraction as *f*′, as it parallels the meaning of *f* from the sequence-structure mapping method, but is based on counting only actual reported structures. By this method, *Pyrobaculum aerophilum* stands on top with *f*′ = 0.7; this value is about twice the value of *f* predicted from sequence-structure mapping. *Sulfolobus solfataricus*, *Pyrococcus abyssi*,* Pyrococcus furiosus*, and the bacteria *Thermotoga Maritima* give values for *f*′ of 0.37, 0.23, 0.23, and 0.091, respectively. Aside from *Pyrobaculum, *where the relatively low number of unique reported structures (25) could explain the anomalously high value of *f*′, the values of *f*′ are well correlated with the estimates, *f*, obtained by the genomic sequence-structure mapping approach (Figure 5). This high correlation provides additional support for using the value of *f* as an indicator of disulfide richness in sequenced genomes.

## 3. Conclusions

Computational analysis of the growing database of sequenced genomes shows convincingly that protein disulfides are ubiquitous among the thermophilic archaea. This is especially true of the Crenarchaea, which are practically all hyperthermophilic, and which appear to be universally rich in disulfides. Disulfide bonding is less abundant and more variable across the Euryarchaea, appearing mainly in the nonmethanogenic thermophiles. Based on the single genome sequence presently available, the ancient Nanoarchaeal branch also appears to be hyperthermophilic and disulfide rich.

In multiple studies, experiments on disulfide bonded proteins from thermophilic microbes have confirmed, as expected, that those disulfide bonds play a major role in stabilizing the folded structure against unfolding and aggregation [22–26]. Proteins and enzymes derived from thermophiles have already been recognized for their utility in industrial applications [8, 49]. Specific proteins or specific homologues that have one or multiple predicted disulfide bonds could make especially attractive choices for such applications, especially since ambient conditions are generally oxidizing. The growing list of disulfide-rich organisms will continue to increase the availability of homologous enzymes for this purpose.

Finally, accelerating the acquisition of genomic data within the archaea, particularly along the Crenarchaeal branch, could have a major impact on the long-standing problem of predicting three-dimensional protein structures from sequence data. Except for a rather narrow target group—for example, very small, mainly alpha helical proteins—accurate *de novo* protein structure prediction remains unreliable [50]. However, additional information in the form of even a few spatial constraints could push structure prediction algorithms over the current barrier. Crenarchaeal proteins represent cases where such spatial constraints might reasonably be inferred from sequence data. For a protein with homologues among the Crenarchaea, a correctly predicted structure will tend to place cysteine residues in proximity for disulfide bonding, whereas no such tendency would be expected for an incorrect structure prediction. Research along this line is presently underway in our laboratory.

## 4. Materials and Methods

### 4.1. Proteome Datasets

The complete proteomes used in this analysis were extracted from UniProtKB release 2011_04. A query with the keyword “complete proteome” returned 90 archaeal proteomes gathering 213232 protein sequences and stocked in FASTA format to constitute the dataset. For metagenomic data from hot springs in Yellowstone National Park, sequences of cellular DNA were included from the most recent samples at two locations, Crater Hills (1152 protein sequences from sample ID CH0909) and Nymph Lake site 10 (1762 protein sequences from sample ID NL10_0908), along with viral DNA sequences (total of 161 protein sequences from four sites: CH, NL10, NL17, and NL18). Contigs were assembled with Newbler gsAssembler v 2.3 (98% nucleotide identity and 50 bp overlap) and translated on-the-fly in the structure-mapping procedure by using Blastx. Sources for the DNA sequences are described in [38].

### 4.2. Filtering Extracytoplasmic and Metalloproteins

As a first step, protein sequences that did not contain at least two cysteines were excluded from the analysis. Moreover, to substantially avoid a biased counting due to cysteines that could be involved in metal binding sites, cysteines falling within 5 residues of each other were not considered, based on the observation that metal binding sites are often (though not always) formed by residues closely spaced in sequence. We also filtered to remove secreted or periplasmic proteins as these are outside the scope of our study; it is already recognized that proteins in these compartments are often rich in disulfides. The PREDISI program was used to perform this filtering step, employing default parameters [51].

### 4.3. Sequence-Structure Mapping

Each protein from the dataset was processed to match it to a homologous structure in the PDB [52]. Pairwise alignment between a UniProtKB sequence and a PDB sequence was performed by BLAST. If a hit with an *E*-value < 0.0001 was found, the sequence was mapped onto the structure. Numerous discrepancies were noted between the PDB sequences appearing in the BLAST database and the sequences reported in the corresponding PDB entry. Therefore, in order to obtain a correct mapping, the positions of the mapped residues were recalculated via a full dynamic programming alignment between the two sequences using the Needleman and Wunsch algorithm [53].

### 4.4. Calculation of the Disulfide-Bond Richness Parameter, *f*

A pair of cysteine residues is judged to represent a probable disulfide bond if their C-alpha atoms are spatially closer than 8 Å when they are mapped onto a homologous structure. In this case, each of the participating cysteines will be considered as a hit; if a given cysteine is within the cutoff distance of more than one other cysteine, it is only counted once. For each protein, the fraction of predicted cysteines forming disulfide bonds can be calculated. Subsequently, at the proteome level, *f* will stand for the ratio between the total number of hits and the total number of mapped cysteines.

### 4.5. Strain Filtering

In our reporting, the redundancy due to multiple sequencing of similar strains of the same species has been removed. For a given species, multiple strain variations were removed if they showed similar results (e.g., a number of mapped proteins within +/− 10% of the parent strain).

### 4.6. Control Datasets

Protein structures of selected species were extracted from the Protein Data Bank (PDB). Cysteine residues involved in disulfide bonds were deduced from their presence in the SSBOND record in the PDB entry while those found in the LINK record were not taken into account, as these typically represent cysteines involved in binding metals or other ligands. Proteins with Zn or Fe bound were excluded from the analysis. Based on the extracted PDB files for a specific organisms, the fraction of cysteines involved in disulfide bonds, *f*′, was calculated based on the same principle as that used to calculate *f* in the sequence-structure mapping approach.

### 4.7. Phylogenetic Analysis

The phylogenetic tree (Figure 3) was built with the web-based tool ITOL [54], after modifying the raw postscript output to illustrate the temperatures and computed disulfide parameters as lines of variable thickness. Optimal growth temperatures used for the circular plot were retrieved from the German Resource Centre for Biological Material website (http://www.dsmz.de/) and from the literature.

## Supplementary Material

The predicted protein disulfide abundance is shown for archaeal microbes with complete genome sequences. A table and figure relate those values to optimum growth temperature and organism phenotypes.
