# Supplementary material for: Widespread Disulfide Bonding in Proteins from Thermophilic Archaea
Source: Archaea. 2011 Sep 20;2011:409156. doi: 10.1155/2011/409156 (PMC3177088; doi:10.1155/2011/409156)
Supplement: Supplementary file 1 — The predicted protein disulfide abundance is shown for archaeal microbes with complete genome sequences. A table and figure relate those values to optimum growth temperature and organism phenotypes. [file 409156.f1.pdf]

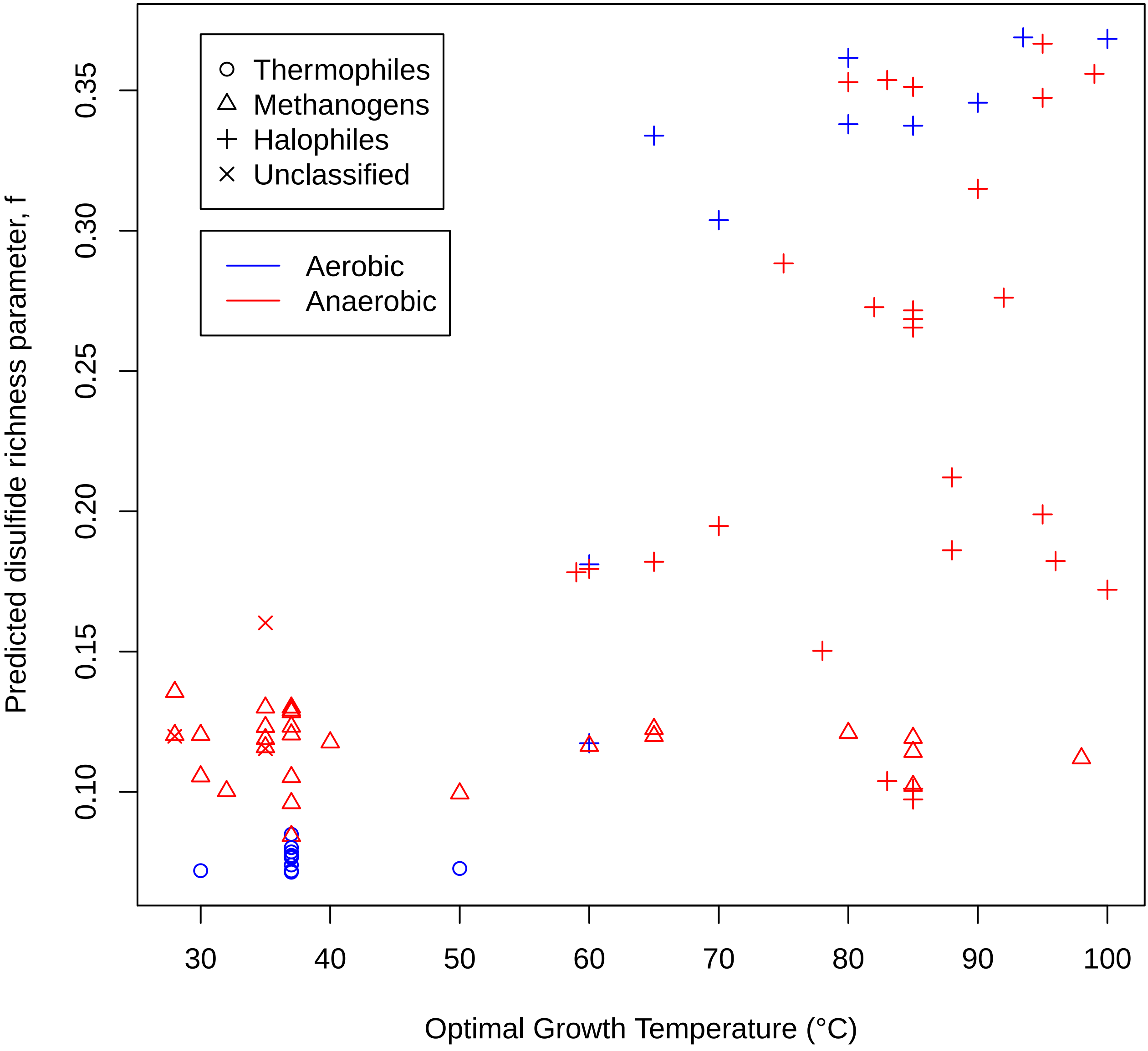
Thermophiles
Methanogens
Halophiles
Unclassified
Aerobic
Anaerobic
Predicted disulfide richness parameter, f
0.10
0.15
0.20
0.25
0.30
0.35
30
40
50
60
70
80
90
100
Optimal Growth Temperature (°C)

Table

OrganismID	Organism Name	Mapped Cysteines	fraction f	OGT
56636	Aeropyrum pernix	835	0.369	93.5
178306	Pyrobaculum aerophilum str. IM2	1200	0.368	100
340102	Pyrobaculum arsenaticum DSM 13514	1192	0.367	95
273063	Sulfolobus tokodaii str. 7	1239	0.362	80
415426	Hyperthermus butylicus DSM 5456	843	0.356	99
397948	Caldivirga maquilingensis IC-167	1148	0.354	83
666510	Acidilobus saccharovorans 345-15	748	0.353	80
444157	Thermoproteus neutrophilus V24Sta	968	0.351	85
384616	Pyrobaculum islandicum DSM 4184	1048	0.347	95
410359	Pyrobaculum calidifontis JCM 11548	1114	0.346	90
427317	Sulfolobus islandicus M.14.25	1376	0.338	80
555311	Sulfolobus solfataricus 98/2	1310	0.337	85
399549	Metallosphaera sedula DSM 5348	1249	0.334	65
453591	Ignicoccus hospitalis KIN4/I	851	0.315	90
2285	Sulfolobus acidocaldarius	1205	0.304	70
228908	Nanoarchaeum equitans Kin4-M	163	0.288	75
583356	Ignisphaera aggregans DSM 17230	1235	0.276	92
490899	Desulfurococcus kamchatkensis 1221n	858	0.273	82
399550	Staphylothermus marinus F1	799	0.272	85
591019	Staphylothermus hellenicus DSM 12710	797	0.269	85
633148	Thermosphaera aggregans DSM 11486	840	0.265	85
368408	Thermofilum pendens Hrk 5	1061	0.212	88
70601	Pyrococcus horikoshii OT3	915	0.199	95
439481	Aciduliprofundum boonei T469	1294	0.195	70
593117	Thermococcus gammatolerans EJ3	994	0.186	88
272844	Pyrococcus abyssi GE5	1015	0.182	96
69014	Thermococcus kodakarensis KOD1	956	0.182	65
273116	Thermoplasma volcanium GSS1	1121	0.181	60
523850	Thermococcus onnurineus NA1	964	0.179	60
273075	Thermoplasma acidophilum DSM 1728	1105	0.178	59
186497	Pyrococcus furiosus DSM 3638	1052	0.172	100
374847	Candidatus Korarchaeum cryptofilum OPF8	1192	0.160	85
604354	Thermococcus sibiricus MM 739	1118	0.150	78
456442	Methanoregula boonei 6A8	3333	0.136	28
2209	Methanosarcina mazei	3944	0.130	35
323259	Methanospirillum hungatei JF-1	4021	0.130	37
269797	Methanosarcina barkeri str. Fusaro	4410	0.129	37
304371	Methanocella paludicola SANAE	3504	0.128	37
410358	Methanocorpusculum labreanum Z	2815	0.123	37
2214	Methanosarcina acetivorans	4864	0.123	35
187420	Methanothermobacter thermautotrophicus s	2148	0.122	65
579137	Methanocaldococcus vulcanius M7	2289	0.121	80
419665	Methanococcus aeolicus Nankai-3	2349	0.120	37
259564	Methanococcoides burtonii DSM 6242	2901	0.120	20
521011	Methanosphaerula palustris E1-9c	3400	0.120	28
79929	Methanothermobacter marburgensis str. Ma	2110	0.120	65

436308	Nitrosopumilus maritimus SCM1	1978	0.120	28
2190	Methanocaldococcus jannaschii	2197	0.119	85
368407	Methanoculleus marisnigri JR1	3155	0.119	25
426368	Methanococcus maripaludis C7	2533	0.118	40
263820	Picrophilus torridus DSM 9790	1210	0.117	60
349307	Methanosaeta thermophila PT	2397	0.116	60
547558	Methanohalophilus mahii DSM 5219	2777	0.116	35
46770	Cenarchaeum symbiosum	1872	0.115	35
573063	Methanocaldococcus infernus ME85	2083	0.114	85
2320	Methanopyrus kandleri	1768	0.112	98
406327	Methanococcus vannielii SB	2416	0.106	30
634498	Methanobrevibacter ruminantium M1	2346	0.105	37
2234	Archaeoglobus fulgidus	2600	0.104	83
573064	Methanocaldococcus fervens AG86	2046	0.102	85
572546	Archaeoglobus profundus DSM 5631	2146	0.101	85
456320	Methanococcus voltae A3	2254	0.100	32
644295	Methanohalobium evestigatum Z-7303	2403	0.099	50
589924	Ferroglobus placidus DSM 10642	2333	0.097	85
420247	Methanobrevibacter smithii ATCC 35061	2125	0.096	37
485914	Halomicrobium mukohataei DSM 12286	2332	0.085	37
339860	Methanosphaera stadtmanae DSM 3091	1922	0.084	37
2238	Haloarcula marismortui	3019	0.080	37
547559	Natrialba magadii ATCC 43099	2531	0.079	37
543526	Haloterrigena turkmenica DSM 5511	2842	0.077	37
795797	Halalkalicoccus jeotgali B3	2989	0.077	37
519442	Halorhabdus utahensis DSM 12940	1869	0.077	37
348780	Natronomonas pharaonis DSM 2160	2217	0.074	37
2242	Halobacterium salinarum	1650	0.073	50
416348	Halorubrum lacusprofundi ATCC 49239	2377	0.072	30
362976	Haloquadratum walsbyi DSM 16790	2087	0.072	37
309800	Haloferax volcanii DS2	2941	0.071	37
